# Case of Concealed Delivery

**Published:** 1847-01

**Authors:** S. C. Sewell


*Case af Concealed Delivery.* By S. C. Sewell, M. D.—On the 16th of November, 1845, at a quarter past 1, P. M., I was called to Mr. K.'s to see his servant Bridget Cloone, æt. 40, who was said to be suffering from colic and pain in the back. Laying my hand on the abdomen, I perceived that she was about seven or eight months pregnant. On my charging her with the fact, she denied it stoutly, said she had menstruated two months before, and finally, finding that she made no impression on my opinion, she declared, in the most solemn manner, "that whatever was inside of her, it was no child." The reason for this statement will appear presently. I had her removvd to the University Lying-in Hospital, whither I

went in an hour after, and finding that the physician in ordinary had not arrived, at the matron's request I examined her *per vaginam*, and found the os uteri dilated and the membranes protruding; presently I detected what appeared to be a cord lying coiled in the upper part of the vagina, and on pulling at it a free extremity came down, but not the os externum. There were no clots of blood in the vagina. At half past five I returned, and found Dr. McCulloch in attendance; the child just being born by the feet, and the woman still persisting that there was no child. The child was feeble, but not at all exsanguined. It survived a few hours. To the placenta were attached two cords; that of the first child had evidently been divided with scissors, from the appearance of the cut surface. Information was given at the police office, that a new-born child had been concealed, for the woman denied that any previous birth had taken place. On searching the bed-room she had occupied at her master's house, the bed bore evident marks of a delivery having taken place, and on searching her trunk the body of a male child was found, underneath the clothes, which had been very carefully smoothed over it. Care was taken not to disturb the position of the limbs, and the body was removed to the Police Station House.

An inquest was held on the following day, when Dr. McCulloch and I were directed to perform the autopsy, of which the following is the result:—The body was fifteen inches long, and weighed two pounds fifteen ounces avoirdupois. The body was not exsanguined; there was no fracture of the skull; the conjunctiva was intensely injected; the cornea hazy and pupil open.

The body was found on its right side in the box, and was deposited on the same side in the station house; in consequence, livid patches were observed on that side from the gravitation of the blood.

*External Examination.*—Several marks of injury were found as follows:—One from the right nipple to the point of the shoulder, half an inch broad; one from the right side of the hyoid bone to mastoid process of right temporal bone; one a little lower, and to the outside, which terminated at the back of the neck; the fourth commencing to the outside again, went to the middle of the superior costa of the scapula; the hands were turned up to the head, the right one to the right ear. The nails were formed. The umbilical cord had been divided nine inches from the body, evidently with scissors, and there was no ligature on it. Meconium was protruding from the anus; the testicles had descended; the thighs were flexed on the abdomen, and the legs on the thighs.

*Internal Examination.*—The marks of injury before referred to were cut into, and the cellular tissue underneath was found to be red with extravasated blood. An incision was made through the lower lip, and down to the epigastrium, in the mesial line. On dividing the lower lip, the tongue was found protruded more than a line beyond the gums. On opening the thorax, the following observations were made:—The apex of the diaphragm was opposite the fifth rib; the lateral portions were well descended; the lungs were of a uniform bright scarlet color, occupying the lateral portions of the thorax, and touching the diaphragm below, but not filling the pleural cavities entirely. The heart and great vessels were nearly in the mesial line, and the cavity of the entire thorax was large for the size of the child; the lungs crepitated on pressure; the lungs, heart, and thymus gland were then removed, and, on being put into water, floated; crepitation occurred under the scalpel; a portion of the lung was squeezed under water, and bubbles issued from every part of the cut surface; the same was observed on squeezing a portion in air; nearly half of each lung was removed, and the remainder, with the heart and thymus still attached, was cast into water, when the mass again floated; portions of the lung floated in water, the cavities of the heart contained dark blood, slightly coagulated; foramen ovale was closed, but not obliterated.

*Inferences.*—1st. The child had breathed freely.

2d. The marks of injury on the right breast and neck were inflicted during life.

3d. They were in all probability occasioned by the left hand of an adult grasping the neck of the infant.

4th. The protrusion of the tongue, and position of the hands, are, probably, referable to strangulation.

5th. Death was not caused by hemorrhage from the cord.

6th, The child was between seven and eight months of utero-gestation,

The rest of the evidence went to show that Bridget Cloone had been a widow for some years; that she had carefully concealed her pregnancy; that she had taken powerful emmenagogue medicines, prescribed by an irregular practitioner, up to the day of delivery, and that she was seen, half an hour before my arrival, to get out of bed, stand by its side, take a pair of scissors from under the pillow, and cut something under the bedclothes.

The coroner's jury brought in a verdict of "wilful murder." The bill of indictment founded thereon was thrown out by the Grand Jury. She was then indicted for concealing the birth of an illegitimate child, convicted, and sentenced to six month's imprisonment.

The above is an exceedingly important case in the annals of medical jurisprudence; and cases of the kind are very rare. Under the hope of escaping from the consequences incident to an actual infanticide, of which there is the strongest probability, this woman persisted *to the last that she was not pregnant*, little anticipating that a second child was to furnish its quota of evidence of the birth of a former one a few hours previously. The case furnishes a striking proof of the fact, that a woman may be delivered of one child, of which she may criminally dispose, for the purpose of concealing its birth, and may afterwards be delivered of a second, the life of which may be preserved.—*British Am. Jour. Med.*

